# Supplementary material for: Witnessing their mother’s acute and prolonged stress affects executive functioning in children
Source: Commun Psychol. 2024 Oct 23;2:98. doi: 10.1038/s44271-024-00150-0 (PMC11500099; doi:10.1038/s44271-024-00150-0)
Supplement: Supplementary file 2 — Supplementary Information [file 44271_2024_150_MOESM2_ESM.pdf]

# Supplementary Information

## Witnessing Their Mother's Acute and Prolonged Stress Affects Executive Functioning in Children

Eileen Lashani[1,2,3,4*], Isabella G. Larsen[5*], Philipp Kanske[6], Jenny Rosendahl[1], Jost U. Blasberg[1†], Veronika Engert[1,2,3,7†]

[1]Institute of Psychosocial Medicine, Psychotherapy and Psychooncology, Jena University Hospital, Jena, Germany

[2]German Center for Mental Health (DZPG), partner site Halle-Jena-Magdeburg, Germany

[3]Center for Intervention and Research on adaptive and maladaptive brain Circuits underlying mental health (C-I-R-C), Jena-Magdeburg-Halle, Germany

[4]Department of Psychiatry and Psychotherapy, Jena University Hospital, Jena, Germany

[5]Department of Psychology and Neuroscience, Department of Public Policy Studies, Duke University, Durham, NC, United States

[6]Clinical Psychology and Behavioral Neuroscience, Faculty of Psychology, Technische Universität Dresden, Dresden, Germany

[7]Social Stress and Family Health Research Group, Max-Planck-Institute for Human Cognitive and Brain Sciences, Leipzig, Germany

### Author Note

[*] These authors contributed equally.

[†] These authors jointly supervised this work.

Correspondence concerning this article should be addressed to Eileen Lashani, Institute of Psychosocial Medicine, Psychotherapy and Psychooncology, Stoystraße 3, 07743 Jena, Germany. Email: Eileen.Lashani@med.uni-jena.de

**Supplementary Table 1**

*Means, standard deviations, and correlations of predictor variables and children's cognitive performance*

| | *M* | *SD* | 1 | 2 | 3 | 4 | 5 | 6 | 7 | 8 | 9 | 10 | 11 | 12 | 13 | 14 | 15 | 16 |
|---|---|---|---|---|---|---|---|---|---|---|---|---|---|---|---|---|---|---|
| 1. Child age | 10.00 | 1.40 | | | | | | | | | | | | | | | | |
| 2. Mother age | 40.36 | 3.26 | .35** | | | | | | | | | | | | | | | |
| 3. Child SS | 13.76 | 63.26 | .23* | -.00 | | | | | | | | | | | | | | |
| 4. Child HR | 64.51 | 706.49 | -.09 | -.16 | .32** | | | | | | | | | | | | | |
| 5. Child CORT | -24.17 | 38.35 | .20 | -.10 | .28* | .00 | | | | | | | | | | | | |
| 6. Mother SS | 24.52 | 70.48 | .04 | .06 | -.10 | -.06 | .10 | | | | | | | | | | | |
| 7. Mother HR | 65.91 | 444.16 | -.20 | -.11 | .13 | .13 | -.11 | .34** | | | | | | | | | | |
| 8. Mother CORT | 17.07 | 53.20 | .01 | -.12 | -.02 | .07 | .12 | .46** | .31** | | | | | | | | | |
| 9. PSS-C | 9.79 | 4.56 | -.03 | -.15 | .03 | .12 | .13 | .17 | -.05 | .00 | | | | | | | | |
| 10. PSS | 14.62 | 4.96 | -.02 | .17 | .13 | .15 | .07 | -.01 | .00 | -.12 | .13 | | | | | | | |
| 11. Child IOS | 5.84 | 1.12 | -.12 | .03 | -.19 | -.31** | -.12 | .19 | .13 | .12 | -.16 | -.24* | | | | | | |
| 12. Mother IOS | 4.55 | 1.36 | -.30** | -.12 | -.08 | -.13 | -.15 | -.09 | .03 | .03 | -.17 | .06 | .02 | | | | | |
| 13. EmQue-CA | 6.20 | 1.79 | .27* | .09 | .00 | .07 | .08 | -.11 | -.16 | -.04 | -.23* | -.01 | -.05 | -.23 | | | | |
| 14. Switch RT | 1398.25 | 264.24 | -.25* | -.22 | -.07 | -.04 | -.11 | -.12 | -.00 | .15 | -.13 | -.12 | .17 | .32** | -.37** | | | |
| 15. Switch ER | 0.21 | 0.14 | -.49** | -.23* | -.21 | -.02 | .06 | .12 | .23* | .30* | .05 | -.07 | .18 | .17 | -.14 | -.01 | | |
| 16. n-back RT | 693.99 | 204.87 | -.24* | -.20 | -.07 | -.01 | .03 | -.31** | -.15 | -.00 | -.15 | .07 | -.13 | .21 | -.07 | .50** | -.08 | |
| 17. n-back d‘ | 1.81 | 1.16 | .14 | .10 | .01 | .04 | .02 | .04 | .03 | .08 | .04 | .12 | -.02 | -.05 | -.03 | .04 | -.38** | .09 |

*Note. M* and *SD* represent mean and standard deviation. SS, HR, and CORT represent the $AUC_i$ of subjective stress, heart rate, and cortisol, respectively. The PSS is the Perceived Stress Scale (Cohen et al., 1983) and the PSS-C the child version (White, 2014). IOS stands for the Inclusion of Other in the Self Scale (Aron et al., 1992) and EmQue-CA for the cognitive scale of the Empathy Questionnaire for Children and Adolescents (Overgaauw et al., 2017). As a measure of speed, mean reaction times (RT) are indicated in milliseconds for the category-switching task (switch; Mayr & Kliegl, 2000) and the n-back task (n-back; Kirchner, 1958). As a measure of accuracy, error rates (ER) and a d'-index are reported. * indicates $p < .05$. ** indicates $p < .01$.

**Supplementary Table 2**

*ANOVA results for comparisons in cognitive performance and perceived stress between control and stress groups*

| | Control Group | Stress Group | | | | |
|---|---|---|---|---|---|---|
| | *M* (*SD*) | *M* (*SD*) | *F*-value | *p*-value | Cohen's *d* | 95% *CI* |
| Category-switching task | | | | | | |
| Reaction time | 1243.11 (247.85) | 1215.76 (274.46) | *F*(1,73) = 0.28 | .599 | -0.1 | -0.33, 0.12 |
| Error rate | 0.15 (0.1) | 0.18 (0.16) | *F*(1,73) = 1.37 | .245 | 0.24 | 0.01, 0.47 |
| N-back task | | | | | | |
| Reaction time | 664.05 (204.24) | 656.64 (162.17) | *F*(1,72) = 0.03 | .853 | -0.04 | -0.36, 0.28 |
| d' | 2.08 (1.13) | 2.18 (1.31) | *F*(1,70) = 0.33 | .565 | 0.08 | -0.25, 0.4 |
| Perceived stress | | | | | | |
| PSS | 14.66 (5.08) | 14.44 (5.03) | *F*(1,72) = 0.04 | .851 | -0.04 | -0.5, 0.41 |
| PSS-C | 10.37 (4.55) | 9.46 (4.68) | *F*(1,70) = 0.72 | .400 | -0.2 | -0.66, 0.26 |

*Note.* Means (M) and standard deviations (*SD*) are presented for each experimental group for a given outcome. Outcomes comprise speed and accuracy on the category-switching task (Mayr & Kliegl, 2000) and the n-back task (Kirchner, 1958), as well as on the PSS (Perceived Stress Scale; Cohen et al., 1983) and the PSS-C (Perceived Stress Scale for Children; White, 2014). Statistical results were obtained using ANOVAs. Cohen's *d* and the respective 95% confidence intervals (*CI*) are reported as an effect size of the difference between the two means.

**Supplementary Table 3**

*Linear mixed model results for a) linear and b) quadratic effects of acute stress on children's reaction time in the category-switching task*

| | Subjective stress | | | | | | Heart rate | | | | | | Cortisol | | | | | |
|---|---|---|---|---|---|---|---|---|---|---|---|---|---|---|---|---|---|---|
| | β | *SE* | $CI_{lower}$ | $CI_{upper}$ | *t*-value | *p*-value | β | *SE* | $CI_{lower}$ | $CI_{upper}$ | *t*-value | *p*-value | β | *SE* | $CI_{lower}$ | $CI_{upper}$ | *t*-value | *p*-value |
| **a)** | | | | | | | | | | | | | | | | | | |
| Intercept | 1004.82 | 44.95 | 915.75 | 1091.76 | 22.35 | **< .001** | 1033.5 | 44.16 | 944.18 | 1118.25 | 23.4 | **< .001** | 1051.23 | 46.82 | 961.55 | 1142.51 | 22.45 | **< .001** |
| Age | -52.1 | 24.85 | -100.51 | -3.65 | -2.1 | **0.037** | -57.12 | 25.96 | -108.92 | -9.11 | -2.2 | **0.029** | -50.9 | 24.63 | -98.17 | -2.97 | -2.07 | **0.04** |
| Sex | 131.25 | 50.14 | 38.61 | 230.93 | 2.62 | **0.009** | 133.43 | 48.9 | 38.76 | 227.47 | 2.73 | **0.007** | 127.76 | 48.79 | 35.02 | 227.69 | 2.62 | **0.009** |
| CTI | 128.09 | 12.68 | 104.73 | 154.4 | 10.1 | **< .001** | 128.09 | 12.68 | 104.73 | 154.4 | 10.1 | **< .001** | 128.09 | 12.68 | 104.73 | 154.4 | 10.1 | **< .001** |
| Switch | 164.42 | 12.62 | 140.2 | 189.04 | 13.03 | **< .001** | 164.42 | 12.62 | 140.2 | 189.04 | 13.03 | **< .001** | 164.42 | 12.62 | 140.2 | 189.04 | 13.03 | **< .001** |
| Condition | 23.94 | 59.21 | -90.61 | 141.4 | 0.4 | 0.686 | -31.82 | 54.4 | -135.55 | 82.37 | -0.58 | 0.559 | -67.54 | 56.31 | -172.96 | 45.37 | -1.2 | 0.231 |
| Child $AUC_i$ | 3.4 | 28.56 | -52.53 | 60.17 | 0.12 | 0.905 | -21.17 | 24.83 | -68.84 | 26.69 | -0.85 | 0.395 | -37.69 | 24.46 | -83.47 | 9.58 | -1.54 | 0.125 |
| Mother $AUC_i$ | -45.76 | 29.66 | -105.67 | 11.59 | -1.54 | 0.124 | -0.93 | 26.92 | -51.64 | 51.8 | -0.03 | 0.972 | 36.78 | 28.21 | -18.93 | 90.63 | 1.3 | 0.193 |
| Child*mother $AUC_i$ | -23.88 | 23.87 | -70.12 | 22.42 | -1 | 0.318 | 2.5 | 20.15 | -36.92 | 42.22 | 0.12 | 0.901 | 32.98 | 24.99 | -17.37 | 81.48 | 1.32 | 0.188 |
| **b)** | | | | | | | | | | | | | | | | | | |
| Intercept | 1011.53 | 46.25 | 922.97 | 1100.3 | 21.87 | **< .001** | 1025.91 | 51.76 | 924.7 | 1132.72 | 19.82 | **< .001** | 1050.59 | 60.15 | 940.73 | 1170.29 | 17.47 | **< .001** |
| Age | -52.39 | 25.17 | -101.36 | -2.13 | -2.08 | **0.038** | -60.22 | 27 | -114.99 | -12.85 | -2.23 | **0.026** | -52.01 | 25.07 | -100.71 | -4.59 | -2.07 | **0.039** |
| Sex | 130.91 | 51.08 | 33.78 | 230.14 | 2.56 | **0.011** | 133.95 | 50.09 | 39.26 | 230.57 | 2.67 | **0.008** | 128.2 | 50.68 | 30.96 | 230.05 | 2.53 | **0.012** |
| CTI | 128.09 | 12.68 | 104.73 | 154.4 | 10.1 | **< .001** | 128.09 | 12.68 | 104.73 | 154.4 | 10.1 | **< .001** | 128.09 | 12.68 | 104.73 | 154.4 | 10.1 | **< .001** |
| Switch | 164.42 | 12.62 | 140.2 | 189.04 | 13.03 | **< .001** | 164.42 | 12.62 | 140.2 | 189.04 | 13.03 | **< .001** | 164.42 | 12.62 | 140.2 | 189.04 | 13.03 | **< .001** |
| Condition | 24.15 | 62.1 | -94.4 | 145.13 | 0.39 | 0.698 | -32.33 | 55.24 | -139.2 | 84.59 | -0.59 | 0.559 | -58.11 | 59.28 | -164.86 | 61.76 | -0.98 | 0.328 |
| Child $AUC_i$ | -0.55 | 29.39 | -58.33 | 58.82 | -0.02 | 0.985 | -18.22 | 28.13 | -72.86 | 37.5 | -0.65 | 0.518 | -29.82 | 27.58 | -80.84 | 25.46 | -1.08 | 0.28 |
| Mother $AUC_i$ | -36.45 | 34.11 | -102.56 | 28.84 | -1.07 | 0.286 | -2.15 | 27.32 | -55.38 | 51.3 | -0.08 | 0.937 | 29.69 | 34.61 | -40.82 | 96.93 | 0.86 | 0.392 |
| Child $AUC_i^2$ | -11.13 | 12.91 | -38.36 | 12.29 | -0.86 | 0.389 | -3.87 | 12.27 | -27.56 | 19.81 | -0.31 | 0.753 | -11.58 | 20.3 | -52.54 | 26.54 | -0.57 | 0.569 |
| Mother $AUC_i^2$ | 4.13 | 15.04 | -24.54 | 34.18 | 0.27 | 0.784 | 11.7 | 21.58 | -29.24 | 52.11 | 0.54 | 0.588 | 7.46 | 21.67 | -33.81 | 50.83 | 0.34 | 0.731 |
| Child*mother $AUC_i$ | -31.05 | 26.6 | -84.19 | 17.89 | -1.17 | 0.244 | 0.88 | 21.25 | -43.74 | 41.96 | 0.04 | 0.967 | 29.78 | 27 | -24.31 | 83.08 | 1.1 | 0.271 |

*Note.* All numeric predictors were scaled prior to analysis. The β-values represent the standardized fixed effects predicting reaction time (in milliseconds) in the category-switching task (Mayr & Kliegl, 2000). Fixed effects of interest include the $AUC_i$s (Area Under the Curve with respect to increase) of children's and mothers' stress markers. Standard errors (*SE*), 95% confidence intervals (*CI*), *t*-values, and *p*-values are reported. All values were obtained using bootstrapping with 1000 iterations. Random intercepts were specified for participants. Results reaching the significance threshold of $p < .05$ were marked in bold.

**Supplementary Table 4**

*Linear mixed model results for a) linear and b) quadratic effects of acute stress on children's reaction time in the working memory task*

| | Subjective stress | | | | | | Heart rate | | | | | | Cortisol | | | | | |
|---|---|---|---|---|---|---|---|---|---|---|---|---|---|---|---|---|---|---|
| | β | *SE* | $CI_{lower}$ | $CI_{upper}$ | *t*-value | *p*-value | β | *SE* | $CI_{lower}$ | $CI_{upper}$ | *t*-value | *p*-value | β | *SE* | $CI_{lower}$ | $CI_{upper}$ | *t*-value | *p*-value |
| **a)** | | | | | | | | | | | | | | | | | | |
| Intercept | 576.77 | 34.17 | 510.61 | 646.13 | 16.88 | **< .001** | 598.45 | 33.65 | 532.32 | 664.79 | 17.79 | **< .001** | 610.72 | 37.17 | 537.35 | 680.45 | 16.43 | **< .001** |
| Age | -42.73 | 19.39 | -81.23 | -3.2 | -2.2 | **0.029** | -51.46 | 20.37 | -90.99 | -8.96 | -2.53 | **0.013** | -49.46 | 20.27 | -88.18 | -6.68 | -2.44 | **0.016** |
| Sex | 39.93 | 37.49 | -34.78 | 109.93 | 1.06 | 0.289 | 39.46 | 37.92 | -35.82 | 112.25 | 1.04 | 0.3 | 41.28 | 39.03 | -37.03 | 115.83 | 1.06 | 0.292 |
| n-back | 67.63 | 14.66 | 39.14 | 96.53 | 4.61 | **< .001** | 67.63 | 14.66 | 39.14 | 96.53 | 4.61 | **< .001** | 67.63 | 14.66 | 39.14 | 96.53 | 4.61 | **< .001** |
| Condition | 56.22 | 47.05 | -26.74 | 149.95 | 1.19 | 0.234 | 15.59 | 43.1 | -67.31 | 100.11 | 0.36 | 0.718 | -6.28 | 47.04 | -95.59 | 88.14 | -0.13 | 0.894 |
| Child $AUC_i$ | 7.58 | 21.21 | -33.03 | 50.68 | 0.36 | 0.721 | -5.67 | 19.85 | -43.36 | 33.44 | -0.29 | 0.776 | 22.69 | 20.63 | -18.67 | 62.14 | 1.1 | 0.273 |
| Mother $AUC_i$ | -53.64 | 22.27 | -100.15 | -11.48 | -2.41 | **0.017** | -28.88 | 21.52 | -70.34 | 13.42 | -1.34 | 0.182 | 1.44 | 23.85 | -45.89 | 46.85 | 0.06 | 0.952 |
| Child*mother $AUC_i$ | -14.82 | 18.41 | -49.85 | 21.86 | -0.8 | 0.422 | 6.89 | 16.04 | -24.76 | 37.57 | 0.43 | 0.668 | -9.21 | 19.09 | -45.08 | 29.81 | -0.48 | 0.63 |
| **b)** | | | | | | | | | | | | | | | | | | |
| Intercept | 565.79 | 34.67 | 496.32 | 636.61 | 16.32 | **< .001** | 617.54 | 38.95 | 544.55 | 695.75 | 15.86 | **< .001** | 581.49 | 47.62 | 492.4 | 675.38 | 12.21 | **< .001** |
| Age | -44.2 | 19.67 | -81.68 | -5.04 | -2.25 | **0.026** | -59.98 | 21.11 | -101.85 | -19.41 | -2.84 | **0.005** | -49.13 | 20.45 | -88.31 | -6.46 | -2.4 | **0.018** |
| Sex | 29.56 | 37.44 | -45.01 | 99.87 | 0.79 | 0.431 | 30.1 | 38.38 | -45.45 | 105.59 | 0.78 | 0.434 | 48.41 | 39.91 | -30.29 | 124.45 | 1.21 | 0.227 |
| n-back | 67.63 | 14.66 | 39.14 | 96.53 | 4.61 | **< .001** | 67.63 | 14.66 | 39.14 | 96.53 | 4.61 | **< .001** | 67.63 | 14.66 | 39.14 | 96.53 | 4.61 | **< .001** |
| Condition | 35.43 | 48.05 | -51 | 130.6 | 0.74 | 0.462 | 12.74 | 42.87 | -70.33 | 96.91 | 0.3 | 0.767 | 4.54 | 48.87 | -89.28 | 108.33 | 0.09 | 0.926 |
| Child $AUC_i$ | 11.19 | 21.87 | -29.82 | 55.91 | 0.51 | 0.61 | 11.01 | 22.34 | -33.29 | 56.81 | 0.49 | 0.623 | 25.15 | 21.96 | -18.62 | 68.3 | 1.15 | 0.254 |
| Mother $AUC_i$ | -43.94 | 25.04 | -93.24 | 2.75 | -1.75 | 0.082 | -28.81 | 21.55 | -69.32 | 12.53 | -1.34 | 0.184 | -12.84 | 28.09 | -68.08 | 39.15 | -0.46 | 0.648 |
| Child $AUC_i^2$ | 14.03 | 12.18 | -9.04 | 37.17 | 1.15 | 0.251 | -16.2 | 10 | -36.46 | 2.61 | -1.62 | 0.107 | 4.06 | 17.54 | -32.35 | 35.81 | 0.23 | 0.817 |
| Mother $AUC_i^2$ | 15.15 | 11.24 | -6.5 | 36.17 | 1.35 | 0.18 | 2.73 | 17.73 | -32.48 | 37.39 | 0.15 | 0.878 | 16.79 | 16.99 | -15.21 | 49.95 | 0.99 | 0.325 |
| Child*mother $AUC_i$ | 7.04 | 23.31 | -36.79 | 53 | 0.3 | 0.763 | 10.29 | 16.69 | -22.21 | 41.91 | 0.62 | 0.539 | -14.3 | 20.14 | -51.89 | 28.63 | -0.71 | 0.479 |

*Note.* All numeric predictors were scaled prior to analysis. The β-values represent the standardized fixed effects predicting reaction time (in milliseconds) in the working memory task (n-back task; Kirchner, 1958). Fixed effects of interest include the $AUC_i$s (Area Under the Curve with respect to increase) of children's and mothers' stress markers. Standard errors (*SE*), 95% confidence intervals (*CI*), *t*-values, and *p*-values are reported. All values were obtained using bootstrapping with 1000 iterations. Random intercepts were specified for participants. Results reaching the significance threshold of $p < .05$ were marked in bold.

**Supplementary Table 5**

*Linear mixed model results for a) linear and b) quadratic effects of acute stress on children's accuracy in the working memory task*

| | Subjective stress | | | | | | Heart rate | | | | | | Cortisol | | | | | |
|---|---|---|---|---|---|---|---|---|---|---|---|---|---|---|---|---|---|---|
| | β | *SE* | $CI_{lower}$ | $CI_{upper}$ | *t*-value | *p*-value | β | *SE* | $CI_{lower}$ | $CI_{upper}$ | *t*-value | *p*-value | β | *SE* | $CI_{lower}$ | $CI_{upper}$ | *t*-value | *p*-value |
| **a)** | | | | | | | | | | | | | | | | | | |
| Intercept | 2.34 | 0.22 | 1.92 | 2.8 | 10.63 | **< .001** | 2.17 | 0.22 | 1.75 | 2.61 | 9.89 | **< .001** | 2.22 | 0.24 | 1.76 | 2.7 | 9.41 | **< .001** |
| Age | 0.3 | 0.12 | 0.06 | 0.54 | 2.46 | **0.015** | 0.34 | 0.13 | 0.09 | 0.58 | 2.65 | **0.009** | 0.33 | 0.13 | 0.08 | 0.56 | 2.64 | **0.009** |
| Sex | 0.51 | 0.25 | 0.05 | 1.01 | 2.1 | **0.038** | 0.53 | 0.25 | 0.06 | 1.01 | 2.13 | **0.035** | 0.51 | 0.25 | 0.02 | 1.01 | 1.99 | **0.049** |
| n-back | -0.66 | 0.12 | -0.89 | -0.44 | -5.62 | **< .001** | -0.66 | 0.12 | -0.89 | -0.45 | -5.64 | **< .001** | -0.66 | 0.12 | -0.89 | -0.45 | -5.64 | **< .001** |
| Condition | -0.19 | 0.29 | -0.73 | 0.39 | -0.65 | 0.518 | 0.09 | 0.28 | -0.47 | 0.65 | 0.34 | 0.734 | 0.01 | 0.29 | -0.53 | 0.58 | 0.04 | 0.967 |
| Child $AUC_i$ | -0.04 | 0.14 | -0.31 | 0.23 | -0.28 | 0.781 | 0.06 | 0.13 | -0.21 | 0.31 | 0.47 | 0.639 | -0.01 | 0.13 | -0.24 | 0.26 | -0.07 | 0.941 |
| Mother $AUC_i$ | 0.21 | 0.14 | -0.07 | 0.49 | 1.48 | 0.142 | -0.03 | 0.14 | -0.3 | 0.25 | -0.22 | 0.827 | 0.06 | 0.15 | -0.23 | 0.36 | 0.39 | 0.698 |
| Child*mother $AUC_i$ | 0.17 | 0.12 | -0.05 | 0.38 | 1.49 | 0.138 | 0.04 | 0.1 | -0.16 | 0.22 | 0.36 | 0.721 | 0.02 | 0.12 | -0.2 | 0.26 | 0.2 | 0.841 |
| **b)** | | | | | | | | | | | | | | | | | | |
| Intercept | 2.37 | 0.22 | 1.94 | 2.82 | 10.61 | **< .001** | 2.38 | 0.26 | 1.85 | 2.89 | 9.26 | **< .001** | 2.48 | 0.3 | 1.91 | 3.07 | 8.28 | **< .001** |
| Age | 0.29 | 0.12 | 0.06 | 0.54 | 2.35 | **0.02** | 0.34 | 0.13 | 0.09 | 0.6 | 2.62 | **0.01** | 0.33 | 0.13 | 0.08 | 0.56 | 2.59 | **0.011** |
| Sex | 0.56 | 0.25 | 0.1 | 1.06 | 2.23 | **0.027** | 0.47 | 0.25 | 0 | 0.96 | 1.91 | 0.059 | 0.43 | 0.26 | -0.06 | 0.94 | 1.69 | 0.093 |
| n-back | -0.66 | 0.12 | -0.89 | -0.44 | -5.63 | **< .001** | -0.66 | 0.12 | -0.89 | -0.45 | -5.64 | **< .001** | -0.66 | 0.12 | -0.89 | -0.45 | -5.64 | **< .001** |
| Condition | -0.08 | 0.3 | -0.64 | 0.52 | -0.26 | 0.792 | 0.09 | 0.28 | -0.48 | 0.64 | 0.31 | 0.754 | -0.11 | 0.3 | -0.72 | 0.46 | -0.37 | 0.71 |
| Child $AUC_i$ | -0.03 | 0.14 | -0.31 | 0.26 | -0.18 | 0.856 | 0.1 | 0.15 | -0.2 | 0.39 | 0.66 | 0.51 | -0.06 | 0.14 | -0.32 | 0.22 | -0.41 | 0.685 |
| Mother $AUC_i$ | 0.12 | 0.17 | -0.21 | 0.44 | 0.74 | 0.459 | -0.01 | 0.14 | -0.28 | 0.27 | -0.06 | 0.955 | 0.21 | 0.18 | -0.12 | 0.56 | 1.19 | 0.234 |
| Child $AUC_i^2$ | -0.02 | 0.08 | -0.17 | 0.13 | -0.21 | 0.832 | -0.02 | 0.06 | -0.14 | 0.11 | -0.32 | 0.752 | 0.01 | 0.11 | -0.2 | 0.22 | 0.12 | 0.901 |
| Mother $AUC_i^2$ | -0.1 | 0.07 | -0.23 | 0.04 | -1.29 | 0.198 | -0.17 | 0.11 | -0.37 | 0.05 | -1.53 | 0.129 | -0.18 | 0.11 | -0.39 | 0.03 | -1.66 | 0.1 |
| Child*mother $AUC_i$ | 0.12 | 0.15 | -0.18 | 0.39 | 0.8 | 0.423 | 0.08 | 0.11 | -0.12 | 0.26 | 0.74 | 0.462 | 0.08 | 0.12 | -0.15 | 0.33 | 0.69 | 0.494 |

*Note.* All numeric predictors were scaled prior to analysis. The β-values represent the standardized fixed effects predicting d' in the working memory task (n-back task; Kirchner, 1958). Fixed effects of interest include the $AUC_i$s (Area Under the Curve with respect to increase) of children's and mothers' stress markers. Standard errors (*SE*), 95% confidence intervals (*CI*), *t*-values, and *p*-values are reported. All values were obtained using bootstrapping with 1000 iterations. Random intercepts were specified for participants. Results reaching the significance threshold of $p < .05$ were marked in bold.

**Supplementary Table 6**

*Linear mixed model results for a) linear and b) quadratic effects of chronic stress on children's reaction time and accuracy in the working memory task*

| | Reaction time | | | | | | d' | | | | | |
|---|---|---|---|---|---|---|---|---|---|---|---|---|
| **a)** | β | *SE* | $CI_{lower}$ | $CI_{upper}$ | *t*-value | *p*-value | β | *SE* | $CI_{lower}$ | $CI_{upper}$ | *t*-value | *p*-value |
| Intercept | 606.83 | 33.54 | 541.93 | 674.09 | 18.09 | **< .001** | 2.11 | 0.21 | 1.7 | 2.54 | 9.82 | **< .001** |
| Age | -36.37 | 20.67 | -74.98 | 4.58 | -1.76 | 0.081 | 0.31 | 0.13 | 0.04 | 0.54 | 2.39 | **0.018** |
| Sex | 43.4 | 38.49 | -30.87 | 117.14 | 1.13 | 0.262 | 0.56 | 0.24 | 0.07 | 1.02 | 2.29 | **0.024** |
| n-back | 65.2 | 14.43 | 35.95 | 93.3 | 4.52 | **< .001** | -0.65 | 0.12 | -0.88 | -0.42 | -5.5 | **< .001** |
| Condition | -4.77 | 38.9 | -82.78 | 70.2 | -0.12 | 0.903 | 0.15 | 0.25 | -0.31 | 0.65 | 0.62 | 0.539 |
| PSS-C | -49.15 | 27.64 | -102.17 | 6.17 | -1.78 | 0.078 | 0.01 | 0.17 | -0.33 | 0.34 | 0.06 | 0.952 |
| PSS | 55.01 | 28.05 | 1.19 | 109.06 | 1.96 | 0.052 | 0.13 | 0.18 | -0.21 | 0.47 | 0.73 | 0.467 |
| Condition*PSS-C | 47.03 | 40.13 | -32.18 | 125.32 | 1.17 | 0.243 | 0.08 | 0.24 | -0.39 | 0.55 | 0.32 | 0.753 |
| Condition*PSS | -63.85 | 41.41 | -143.27 | 18.25 | -1.54 | 0.126 | 0.09 | 0.26 | -0.4 | 0.58 | 0.35 | 0.725 |
| PSS-C*PSS | 29.47 | 34.83 | -37.74 | 99.67 | 0.85 | 0.399 | 0.01 | 0.22 | -0.4 | 0.46 | 0.06 | 0.954 |
| Condition*PSS-C*PSS | -27.86 | 45.58 | -119.9 | 63.7 | -0.61 | 0.542 | -0.16 | 0.29 | -0.73 | 0.37 | -0.56 | 0.576 |
| **b)** | | | | | | | | | | | | |
| Intercept | 605.13 | 39.05 | 531.99 | 680.54 | 15.5 | **< .001** | 2.13 | 0.25 | 1.61 | 2.61 | 8.46 | **< .001** |
| Age | -36.17 | 21.35 | -76.34 | 5.94 | -1.69 | 0.093 | 0.3 | 0.13 | 0.04 | 0.54 | 2.27 | **0.025** |
| Sex | 39.75 | 40.18 | -38.59 | 118.81 | 0.99 | 0.324 | 0.54 | 0.26 | 0.02 | 1.04 | 2.09 | **0.038** |
| n-back | 65.2 | 14.43 | 35.95 | 93.3 | 4.52 | **< .001** | -0.65 | 0.12 | -0.88 | -0.42 | -5.5 | **< .001** |
| Condition | -4.1 | 39.49 | -81.73 | 73.3 | -0.1 | 0.918 | 0.16 | 0.25 | -0.3 | 0.65 | 0.64 | 0.523 |
| PSS-C | -45.12 | 31.89 | -103.61 | 18.05 | -1.41 | 0.16 | 0.06 | 0.2 | -0.34 | 0.44 | 0.28 | 0.779 |
| PSS | 55.7 | 28.53 | -0.36 | 110.26 | 1.95 | 0.053 | 0.13 | 0.18 | -0.22 | 0.49 | 0.72 | 0.471 |
| $PSS-C^2$ | -3.15 | 15.53 | -33.59 | 26.24 | -0.2 | 0.839 | -0.04 | 0.1 | -0.25 | 0.15 | -0.44 | 0.658 |
| $PSS^2$ | 5.48 | 15.99 | -25.27 | 37.68 | 0.34 | 0.732 | 0.02 | 0.11 | -0.17 | 0.23 | 0.23 | 0.817 |
| Condition*PSS-C | 41.7 | 43.13 | -41.48 | 125.58 | 0.97 | 0.335 | 0.03 | 0.26 | -0.47 | 0.56 | 0.11 | 0.91 |
| Condition*PSS | -65.7 | 42.51 | -144.79 | 17.67 | -1.55 | 0.125 | 0.09 | 0.26 | -0.4 | 0.59 | 0.33 | 0.743 |
| PSS-C*PSS | 33.15 | 37.07 | -36.44 | 106.16 | 0.89 | 0.373 | 0.05 | 0.23 | -0.38 | 0.53 | 0.22 | 0.827 |
| Condition*PSS-C*PSS | -27.49 | 46.27 | -119.91 | 64.23 | -0.59 | 0.553 | -0.16 | 0.29 | -0.74 | 0.39 | -0.54 | 0.593 |

*Note.* All numeric predictors were scaled prior to analysis. The β-values represent the standardized fixed effects predicting reaction time and d' in the working memory task (n-back task; Kirchner, 1958). Fixed effects of interest include the PSS (Perceived Stress Scale; Cohen et al., 1983) and the PSS-C (Perceived Stress Scale for Children; White, 2014) as markers of children's and mothers' chronic stress. Standard errors (*SE*). 95% confidence intervals (*CI*), *t*-values, and *p*-values are reported. All values were obtained using bootstrapping with 1000 iterations. Random intercepts were specified for participants. Results reaching the significance threshold of $p < .05$ were marked in bold.
