# Supplementary material for: Witnessing their mother’s acute and prolonged stress affects executive functioning in children
Source: Commun Psychol. 2024 Oct 23;2:98. doi: 10.1038/s44271-024-00150-0 (PMC11500099; doi:10.1038/s44271-024-00150-0)
Supplement: Supplementary file 3 — Reporting Summary [file 44271_2024_150_MOESM3_ESM.pdf]

Corresponding author(s): Eileen Lashani

Last updated by author(s): Oct 14, 2024

# Reporting Summary

Nature Portfolio wishes to improve the reproducibility of the work that we publish. This form provides structure for consistency and transparency in reporting. For further information on Nature Portfolio policies, see our Editorial Policies and the Editorial Policy Checklist.

## Statistics

For all statistical analyses, confirm that the following items are present in the figure legend, table legend, main text, or Methods section.

| n/a | Confirmed | |
|---|---|---|
| ☐ | ☒ | The exact sample size ($n$) for each experimental group/condition, given as a discrete number and unit of measurement |
| ☐ | ☒ | A statement on whether measurements were taken from distinct samples or whether the same sample was measured repeatedly |
| ☐ | ☒ | The statistical test(s) used AND whether they are one- or two-sided<br>*Only common tests should be described solely by name; describe more complex techniques in the Methods section.* |
| ☐ | ☒ | A description of all covariates tested |
| ☐ | ☒ | A description of any assumptions or corrections, such as tests of normality and adjustment for multiple comparisons |
| ☐ | ☒ | A full description of the statistical parameters including central tendency (e.g. means) or other basic estimates (e.g. regression coefficient) AND variation (e.g. standard deviation) or associated estimates of uncertainty (e.g. confidence intervals) |
| ☐ | ☒ | For null hypothesis testing, the test statistic (e.g. $F$, $t$, $r$) with confidence intervals, effect sizes, degrees of freedom and $P$ value noted<br>*Give P values as exact values whenever suitable.* |
| ☒ | ☐ | For Bayesian analysis, information on the choice of priors and Markov chain Monte Carlo settings |
| ☐ | ☒ | For hierarchical and complex designs, identification of the appropriate level for tests and full reporting of outcomes |
| ☐ | ☒ | Estimates of effect sizes (e.g. Cohen's $d$, Pearson's $r$), indicating how they were calculated |

*Our web collection on statistics for biologists contains articles on many of the points above.*

## Software and code

Policy information about availability of computer code

| | |
|---|---|
| Data collection | Cognitive tests were conducted in Presentation® 2018. |
| Data analysis | All statistical analyses were conducted using R 4.2.0. Analysis scripts are publicly accessible at https://osf.io/kyrbh/ |

For manuscripts utilizing custom algorithms or software that are central to the research but not yet described in published literature, software must be made available to editors and reviewers. We strongly encourage code deposition in a community repository (e.g. GitHub). See the Nature Portfolio guidelines for submitting code & software for further information.

## Data

Policy information about availability of data

All manuscripts must include a data availability statement. This statement should provide the following information, where applicable:
- Accession codes, unique identifiers, or web links for publicly available datasets
- A description of any restrictions on data availability
- For clinical datasets or third party data, please ensure that the statement adheres to our policy

The data used in this study, including cognitive performance metrics, physiological measures, and questionnaire responses, are publicly accessible at https://osf.io/kyrbh/.

## Human research participants

Policy information about studies involving human research participants and Sex and Gender in Research.

| | |
|---|---|
| Reporting on sex and gender | Our sample included 76 children (n = 37 girls, n = 39 boys) and their mothers . Sex was assessed via self-report and controlled for when pseudo-randomizing the dyads into the experimental groups. All analyses included sex as a covariate. |
| Population characteristics | See below. |
| Recruitment | Participation for this study was promoted online and with posters, targeting schools and after-school care in Leipzig (Germany) and surrounding areas. Mothers underwent a telephone screening before inclusion. Representativeness is limited as only healthy, German, and predominantly highly educated mother-child dyads participated. |
| Ethics oversight | Ethics Committee of University of Leipzig (number: 084/18-ek) |

Note that full information on the approval of the study protocol must also be provided in the manuscript.

# Field-specific reporting

Please select the one below that is the best fit for your research. If you are not sure, read the appropriate sections before making your selection.

☐ Life sciences ☒ Behavioural & social sciences ☐ Ecological, evolutionary & environmental sciences

For a reference copy of the document with all sections, see nature.com/documents/nr-reporting-summary-flat.pdf

# Behavioural & social sciences study design

All studies must disclose on these points even when the disclosure is negative.

| | |
|---|---|
| Study description | quantitative experimental |
| Research sample | The study sample consist of n = 76 children (n = 37 girls, n = 39 boys) and their mothers. Children were 8 to 12 years old (M = 9.95, SD = 1.42) and their mothers were 31 to 45 years old (M = 40.3, SD = 3.33). Representativeness is limited as only healthy, German, and predominantly highly educated mother-child dyads participated. The dyads have previously been described in Blasberg at al. (2023), who investigated a different research question in the same sample. |
| Sampling strategy | The sample size was pre-determined based on a power analysis reported in Blasberg et al. (2023), aiming to detect medium to large effects of empathic stress. |
| Data collection | Questionnaires were administered in pen and paper format. Heart rate was recorded using a wrist blood pressure monitor. Salivary cortisol was collected using Salivettes. Executive functioning was assessed with two computerized tasks testing cognitive flexibility and working memory. Blinding of the experimenters was not possible, as the testing procedure differed between the psychosocial stress test and the stress-free control condition. In the psychosocial stress test, two research assistants in the roles of an evaluation committee were present. |
| Timing | All data was collected between June 2018 and March 2020. |
| Data exclusions | Children with insufficient task understanding were excluded, resulting in the exclusion of one child for the category switching task and two children for the n-back task. Individuals with two or more consecutive missing data points of a stress marker were excluded for the respective analysis, resulting in the exclusion of one mother for cortisol analysis. |
| Non-participation | No participants dropped out or declined participation. |
| Randomization | Mother-child dyads were pseudo-randomly assigned to experimental (n = 39) or control groups (n = 37), controlling for age and sex distribution. |

# Reporting for specific materials, systems and methods

We require information from authors about some types of materials, experimental systems and methods used in many studies. Here, indicate whether each material, system or method listed is relevant to your study. If you are not sure if a list item applies to your research, read the appropriate section before selecting a response.

## Materials & experimental systems

| n/a | Involved in the study |
| --- | --- |
| ☒ | ☐ Antibodies |
| ☒ | ☐ Eukaryotic cell lines |
| ☒ | ☐ Palaeontology and archaeology |
| ☒ | ☐ Animals and other organisms |
| ☒ | ☐ Clinical data |
| ☒ | ☐ Dual use research of concern |

## Methods

| n/a | Involved in the study |
| --- | --- |
| ☒ | ☐ ChIP-seq |
| ☒ | ☐ Flow cytometry |
| ☒ | ☐ MRI-based neuroimaging |
